# Parental education moderates the association between indoor moisture environment and asthma in adolescents: the Greek Global Asthma Network (GAN) cross-sectional study

**DOI:** 10.1186/s12889-022-13065-4

**Published:** 2022-03-28

**Authors:** George Antonogeorgos, Evangelia Liakou, Alexandra Koutsokera, Pavlos Drakontaeidis, Marina Thanasia, Maria Mandrapylia, Sotirios Fouzas, Philippa Ellwood, Luis García-Marcos, Demosthenes B. Panagiotakos, Kostas N. Priftis, Konstantinos Douros

**Affiliations:** 1 Allergology and Pulmonology Unit, 3Rd Paediatric Department, National and Kapodistrian University of Athens, 12462 Athens, Greece; 2 Department of Nutrition and Dietetics, School of Health Sciences and Education, Harokopio University, 17676 Athens, Greece; 3 Division of Paediatrics & Obstetrics – Gynaecology, School of Medicine, University of Patras, Patras, Greece; 4 Department of Paediatrics: Child and Youth Health, Faculty of Medical and Health Sciences, University of Auckland, 1023 Auckland, New Zealand; 5 Paediatric Allergy and Pulmonology Units, ‘Virgen de La Arrixaca’ University Children’s Hospital, University of Murcia, ARADyAL Network and Biomedical Research Institute of Murcia (IMIB-Arrixaca), 30394 Murcia, Spain

**Keywords:** Adolescent, Asthma, Moisture, Parental, Education

## Abstract

**Objective:**

Asthma is a major contributor to childhood morbidity. Several environmental and socioeconomic status (SES) factors have been implicated in its etiopathogeneses such as indoor moisture and parental education level. Our study examined the association between exposure to indoor dampness and/or mould (IDM) with adolescent asthma and how parental education could modify or mediate this relationship.

**Method:**

A total of 1934 adolescents (boys: 47.5%, mean age (standard variation): 12.7(0.6) years) and their parents were voluntarily enrolled and completed a validated questionnaire on adolescents’ asthma status, parental educational level, and adolescents’ indoor exposure to IDM during three different lifetime periods, i.e., pregnancy, the first year of life and the current time.

**Results:**

There was a significant modification effect of parental education only for the current exposure; higher parental education lowered almost 50% the odds of IDM and asthma (adjusted odds ratio (aOR): 1.96, 95% Confidence Intervals (CI): (1.05–3.68) and aOR:1.55, 95% CI (1.04–2.32), for primary/secondary and tertiary parental education, respectively).

**Conclusion:**

Adolescents whose parents had a higher education level had lesser odds to have asthma, even if they were exposed to a moisture home environment. This could be attributed to the increased knowledge about asthma risk factors and the improved measures for the amelioration of moisture-home environment that highly educated parents are more likely to take. Further research is needed in order to elucidate the interweaved role of family SES in the aforementioned relation.

## Background

Asthma is the most common chronic respiratory disease, affecting more than 330 million adults and children of all ages globally. It is estimated that almost one out of five children aged 13–14 years suffers from asthma in English-speaking countries of North America, Europe and Australasia while an estimated 8.6% of the population aged 18–45 years report asthma symptoms (attacks of wheezing or whistling breath) in the past 12 months [1]. In Greece, asthma is a major contributor to total childhood morbidity. Following the global trend, asthma prevalence in Greece has been rising during the last three decades [2]. Current asthma, defined as having any asthma symptoms in the past 12 months, has been reported to be 9% and 5% of the children aged 7 and 18 years, respectively [3].

Several factors have been implicated in the pathogenesis of asthma and its exacerbations. One of them is the exposure to indoor dampness and/or mould (IDM) in various stages of a child’s life. In a meta-analysis of 16 cohort and case–control studies, evidence indicated that IDM was positively associated with the development of asthma in children [4]. There is also evidence for a dose-dependent relationship between the amount of exposure to IDM and asthma prevalence [5]. Another important factor that affects childhood asthma is family socioeconomic status (SES). There is a plethora of evidence connecting low SES and other poverty-related exposures with asthma development and poor asthma outcomes such as low pulmonary function and frequent exacerbations [6–8]. Parental educational level is one of the determinants of SES and also plays an important role per se in childhood asthma. In a study by Gong et al., children with the lowest parental education had an increased likelihood of inpatient and outpatient asthma diagnosis as well as poorer asthma treatment compared to families where at least one parent had a college degree [9].

However, childhood asthma is a multifaceted chronic disease and socioeconomic and home environmental factors could interact in a more complex way with its aetiology. To the best of our knowledge, only one study has reported that the effect of household exposures such as living in a multi-unit dwelling or residence with rodent or insect infestation to childhood asthma is mediated by SES [10]. Thus, in the context of the Greek branch of the Global Asthma Network (GAN) study [11], in order to elucidate the interweaved role of socioeconomic and home environment determinants on childhood asthma we examined whether parental/guardian educational level, as a marker of family SES, is an effect modifier or an effect mediator of the association between asthma in adolescents aged 12–14 years and IDM exposure during three different lifetime periods, i.e., pregnancy, the first year of life and the current time.

## Methods

### Design

This is an observational study and is part of the GAN Phase-I survey. The latter is an international project aimed to monitor the prevalence, severity, management and risk factors of asthma, worldwide [11].

### Setting and sample

The study was conducted in the greater area of Athens, Greece, from February to March 2020, in high-school settings; twenty high-schools (lyceums in Greek terminology) were selected by stratified random sampling from a list containing both public and private schools that was provided by the Secondary Education Office in Athens. High schools were stratified according to the socioeconomical status of all the urban regions of the metropolitan city of Athens. All children in the 1^st^ and 2^nd^ grade of each school were asked to enter in the study. Exclusion criteria for participation in the study were schools for children with special educational needs or disabilities. In total, 2560 child-parent/guardian pairs were asked to participate. Of them, 1,934 adolescents, mean age: 12.7 ± 0.6 years, (921 boys, 47.5% and 1013 girls, 52.5%) and their parents/guardians (25.4% fathers, mean age 49.1 ± 5.5 years, 74.6% mothers, mean age 45.4 ± 4.8 years) agreed to participate (participation rate 76%). Our study sample was powered enough to assess standardized two-sided differences of 10% on the prevalence of ever had asthma between adolescents with different types of exposure to IDM with 95% statistical power at a 5% level of significance. More information could be found elsewhere [12].

### Bioethics

The study was approved by the ethics committee of the National and Kapodistrian University of Athens (decision number: 214/13–12-19). For the execution of the study, permission was issued by the Ministry of Educational Affairs (decision number: 10053/24–01-2020). Informed consent to participate was taken from all subjects/Parents and Legally authorized representatives of minors below age 16 years. The study was conducted in accordance with the guidelines of Declaration of Helsinki.

### Measurements

The GAN study included two standardised questionnaires: one that was completed by the adolescents during schooltime and one intended for parents/guardians to complete at home (adult questionnaire). The adolescent’s questionnaire included several questions assessing symptoms of asthma, rhinitis and eczema, as well as other questions regarding the home environment and lifestyle [11]. Specifically, asthma ever and current asthma was defined as the positive answer to the question “Have you ever had wheezing or whistling in the chest at any time in the past?” and “Have you had wheezing or whistling in the chest in the past 12 months?” respectively. Moreover, adolescents were asked if they had siblings, and if so, how many. Moreover, the height and the weight of the participating children were measured and their body mass index (BMI) was calculated. Parents/guardians were asked to report if they had a history of atopic disease (asthma, eczema or hay fever) and whether and for how long they smoked. Moreover, their educational level was recorded in three categories: primary (compulsory/ 9 years), secondary (non-compulsory/3 years) or tertiary (university/college/post-graduate studies). Due to the small number of parents with primary education (*n* = 24), we merged this level with the next one (secondary education), creating a dichotomous variable (i.e., primary/secondary vs. tertiary parental educational level). For the assessment of the indoor moisture environment, parents/guardians were asked to report if there was visible moisture or mould spots on the walls or ceiling of any room in their homes during three different periods of their child’s life, namely, during pregnancy, the first year of life, and at present.

### Statistical analysis

Continuous and categorical variables are presented as mean ± standard deviation (SD) or absolute and relative frequencies, respectively. The estimation of the odds ratios (OR) and the corresponding 95% confidence intervals (CI) of an adolescent ever having asthma and having current asthma based on the IDM exposure during the three different periods (pregnancy, the first year of its life, and current time exposure) was performed by applying three multiple logistic regression models, respectively. Several well-known from the literature confounders were included in the models, i.e., adolescents’ sex, BMI, parental atopic history, adolescent atopic history (eczema and allergic rhinitis), parental smoking, pet ownership, having an older sibling, cooking with fuels and ventilating the house during cooking. Furthermore, the interaction between IDM exposure with the parental educational level on adolescent’s likelihood of ever having asthma and having current asthma symptoms was tested and the potential mediating effect of parental education was evaluated by Sobel’s test. Deviance residuals were calculated in order to evaluate all logistic models’ goodness-of-fit. All reported probability values (p-values) were based on two-sided tests and compared to a significant level of 5%. STATA 14 software was used for all the statistical calculations (STATA Corp., College Station, TX, USA).

## Results

In Table 1 anthropometric, atopic and lifestyle characteristics of the participating children and their corresponding parent as well as the characteristics of their indoor living environment related to the presence of IDM in the three lifetime periods, are presented. Asthma ever was reported by 8.9% of the adolescents (55.8% boys, *p* = 0.024) and current asthma was 6.2% prevalent in the study sample (44.0% boys, *p *= 0.84). The majority of parents/guardians (66.2%) reported having tertiary education. Regarding the exposure to IDM, 23.7% of the adolescents were reported as currently exposed. Maternal exposure to IDM during pregnancy and exposure of the child during the first year of his/her life were reported at 7.2% and 11.9% of the participants, respectively.

.

In Table 2, the distribution of the investigated factors is presented in relation to parental educational level. Parental smoking was significantly higher among those who had primary or secondary education (*p* < 0.001), while adolescents’ BMI was also significantly higher in this parental education status group (*p* < 0.001). No other significant associations were observed (all p’s > 0.05).

Crude logistic regression analyses revealed that adolescents who were currently exposed to IDM at homeplace were almost 50% and 65% more likely to ever had asthma and having current asthma symptoms as compared to those reporting no exposure adjusted for all confounders (Table 3). However, no significant relationship was revealed for adolescents who were exposed to IDM during their mother’s pregnancy or who were exposed during the first year of their life, either. The test for a potential moderating effect of parental education level on the relationship between adolescents’ exposure to IDM and the presence of asthmatic history and current asthma symptoms showed a significant interaction only between current exposure to IDM (*p* for interaction < 0.001 for both outcomes).

For the completeness of results, the stratified analysis according to parental education for all types of IDM (during pregnancy, during the 1st year of life, and current) is presented in Table 4. Adolescents whose parents had primary or secondary education were almost two-times more likely to ever had asthma or to have any current asthma symptoms if they lived in an environment with IDM compared to the ones not exposed, while these odds lowered by almost 50% when the parental educational level was high (i.e., tertiary) for both asthmatic outcomes.

Furthermore, in order to explore the possible mediating role of parental education, as a SES marker in the aforementioned relationship, Sobel’s test was applied in multiple logistic regression models. In particular, in the first model, adolescents’ exposure to IDM was initially evaluated in relation to asthma symptoms, after adjusting for sex, BMI, parental atopic history and smoking habits, pet ownership, having an older sibling and cooking with fuels. Then, parental education level was entered in the model, and the change in the ORs between the two nested models was evaluated. It was observed that parental education level did not significantly mediate either the effect of the current exposure (p for Sobel test = 0.125) or the effect of exposure during mother’s pregnancy or during the first year of adolescents’ life (p for Sobel test = 0.129 and *p* = 0.326, respectively).

## Discussion

To the best of our knowledge, this study is the first to evaluate the role of parental educational level on the association between exposure to IDM and adolescent asthma. Our results provide evidence that current exposure to IDM is positively associated with asthma in adolescents aged 12–14 years old. However, adolescents at this age group who reside in houses with visible signs of dampness or mould but also have at least one parent of higher education, have an almost 50% reduced odds of ever had asthma or having current asthma symptoms compared to adolescents whose both parents have primary or secondary education. Despite the limitations that arise from the observational design of this study, the findings deserve further attention from a public health perspective, since they demonstrate a moderating, but not a mediating, role of parental education level and family SES on the association of an environmental risk factor such as IDM with childhood asthma.

Exposure to IDM is a long-established risk factor for asthma that has been associated with increased rates of asthma development in childhood, poor asthma control, frequent exacerbations and bad asthma-related quality of life in childhood. This could be attributed to two specific asthmatic endotypes, the high T-helper cell type 2 (Th2) or atopic endotype and the non-high Th2 (or low) endotype. Th2 endotype Immunoglobulin E (IgE) mediated and characterized by high blood/sputum eosinophil count due to allergic sensitization by aeroallergens such as moulds and mites [13]. However, evidence suggest that this association could be weak [14]. Thus, also non-IgE pathophysiological mechanisms could be contributing to the association between IDM and childhood asthma. Our study results are in accordance with the results of previous studies [15, 16]. However, our study showed no significant association between exposure to IDM during pregnancy or during the first year of life with asthmatic history at the age of 12 to 14 years. This finding is in agreement with the results of a meta-analysis that included eight birth cohort studies with 31,742 children and showed that early-life exposure to visible moulds and/or dampness in the first 2 years of life, increased significantly the risk of having asthma at 3 years of age but it had no effect on for later asthma outcomes [17].

Parental education is a major component of the family SES and it is well known that low SES is related to unfavourable outcomes in many diseases, including childhood asthma [18, 19]. There are many studies providing evidence that children originating from low SES backgrounds visit the emergency departments of paediatric hospitals more often and are more likely to be hospitalized for asthma [20, 21]. Moreover, children from families of low SES experience greater limitation in their activities due to asthma [22]. The association of low SES with poor asthma outcomes in childhood could be mediated through the higher levels of chronic stress they suffer, and which is associated with increased production of the cytokines IL-5 and IL-13 as well as with higher eosinophil counts in blood samples, as it was shown in the study by Chen et al. [23]. Furthermore, the effect of low SES during early childhood can affect immune programming, resulting in phenotypes prone to inflammation in mid-life adults [24]. Our study provides further evidence suggesting that family SES, as marked by parental/guardian education level, apart from its independent negative effect on asthma, also interferes with the effect of a poverty-related home-environmental factor such as IDM with asthma. But in contrast with previously reported evidence, no mediating effect was observed [10]. However, when parental/guardian education was tested for possible moderation effect, a significant effect was revealed, proposing a different way that parental/guardian educational level could alter the strength of the aforementioned relationship.

One possible pathway that high parental/guarding education moderates the negative association of exposure to IDM with asthma is through the improved parental knowledge about asthma home-environment related risk factors and all the subsequent necessary actions parents are taking to minimize them. There are a number of studies that support our reported associations. In a study by Kercsmar et al., children in the intervention group who received dampness-related house repairs exhibited significantly fewer asthma exacerbations compared to the control group [25]. Furthermore, in the clinical trial by Krieger et al., 274 low-income children living in an urban environment in Seattle were assigned to two groups: the intervention group who received major in-home environmental assessments related to dampness, and the control group, who received only a limited amount of intervention. Children who belonged to the intervention group had significantly improved asthma-related quality of life scores and less urgent health services use [26]. Finally, in a children-parent clinical study of 150 children aged 9–17 years with physician-diagnosed asthma, higher parental education was associated with better home environment control behaviours [27]. Our evidence expands these previous findings and suggests that highly educated parents/guardians are more likely to understand and intervene with asthma-related home environmental risk factors such as IDM, thus lessening its negative effect on asthma. More focused research is needing, in order to further elucidate the important role of parental education in the association between IDM and asthma in adolescence.

### Limitations

The study has a cross-sectional design, and it suffers the inherited limitations of this type of epidemiological studies, such as recall bias in the requested information about exposure during birth of the first year of life, report bias of measurements and reverse causality bias in the reported associations. However, every effort was made not to over-interpret the study results. Moreover, no information on other SES indicator variables such as household income or health insurance coverage and other environmental factors such as type of housing, family crowding or exposure to termite or cockroaches was available. However, the use of parental education as a SES indicator is very common in adolescent research while all the other major literature-known confounders were included in the multivariate models, thus minimizing the confounding effect.

## Conclusions

Our study expands the current evidence on the association between exposure to IDM and asthma in adolescence and highlights the moderating role of parental education and family SES in this relationship. Thus, adolescents who are exposed to a moisture home-environment but their parents/guardians have higher education are less likely to ever have a diagnosis of asthma. Further research is needed in order to clarify the role of parental education, as a proxy for family SES, in this association. However, public health policymakers should make any effort to ensure that parents have a good knowledge of the role of home-environmental asthma risk factors such as IDM, as an additional way for its prevention.

**Table 1:** Demographic, anthropometric, atopic, lifestyle and indoor environment characteristics of the participating adolescents and their parents/guardians, according to adolescents’ asthmatic status (*n* = 1934)

	**Adolescent’s asthmatic history** **(Ever had asthma)**		***p>***	**Adolescent’s wheezing or whilst in the past 12 months** **(Current asthma)**		*p*
	**Yes**	**No**		**Yes**	**No**	
*N*			-			
*Boys* (n, %)	96 (10.4)	825 (89.6)	0.024	59 (49.2)	862 (47.5)	0.726
Adolescents’ age (years), mean (SD)***	12.8 (0.6)	12.7 (0.6)	0.345	12.7 (0.5)	12.8 (0.7)	0.785
Adolescents’ BMI (kg/m^2^), mean (SD)***	22.3 (4.4)	20.9 (3.4)	< 0.001	21.8 (4.4)	22.4 (4.0)	0.183
Ever had allergic rhinitis (children) (Yes, n, %)	95 (55.2)	472 (26.8)	< 0.001	59 (49.2)	508 (28.0)	< 0.001
Ever had eczema (children) (Yes, n, %)	40 (23.3)	196 (11.1)	< 0.001	24 (20.0)	212 (11.7)	0.007
Pet ownership (Yes, n, %)	120 (8.8)	1250 (91.2)	0.833	64 (53.3)	1040 (57.4)	0.388
Having an older sibling (Yes, n, %)	75 (9.0)	754 (91.0)	0.842	56 (46.7)	773 (42.6)	0.389
Parental age (years), mean (SD)	46.3 (5.1)	46.4 (5.3)	0.0871	46.6 (6.3)	46.5 (5.2)	0.494
Parental atopic history (Yes, n, %)	46 (12.2)	331 (87.8)	0.012	57 (47.5)	1023 (56.3)	0.061
Parental ever smoking (Yes, n, %)	108 (10.0)	972 (90.0)	0.057	42 (35.0)	812 (44.7)	0.038
Parental educational level (Yes, n, %)						
Primary / secondary*	602 (92.3)	50 (7.7)	0.174	45 (37.5)	609 (33.5)	0.374
Tertiary**	1160(90.5)	122 (9.5)		75 (62.5)	1207 (66.5)	
Cooking with fuels (Yes, n, %)	95 (10.1)	842 (89.9)	0.069	62 (51.7)	773 (42.6)	0.289
Current exposure to dampness and/or mould (Yes, n, %)	58 (12.6)	402 (87.4)	0.001	80 (66.7)	1397 (76.9)	0.011
Exposure to dampness and/or mould during pregnancy (Yes, n, %)	11 (7.9)	128 (92.1)	0.672	10 (8.3)	129 (7.1)	0.612
Exposure to dampness and/or mould during the 1^st^ year (Yes, n, %)	26 (11.4)	203 (88.6)	0.164	20 (16.7)	210 (11.6)	0.094

* up to 9 years of education ** university/college/post-graduate studies *** SD: standard deviation

**Table 2:** Children’s anthropometric, parental atopic history and indoor environment characteristics in various exposure times according to parental educational level

	**Parental educational level**		
	Primary or secondary* (***n*** = 652)	Tertiary** (***n ***= 1282)	***p***
Adolescent’s asthmatic history (Yes, n, %)	50 (7.7)	122 (9.5)	0.178
Adolescent’s wheeze in the past 12 months (Yes, n%)	45 (6.9)	75 (5.9)	0.374
Children’s BMI (kg/m^2^)	21.54 (3.72)	20.71 (3.43)	< 0.001
Having an older sibling (Yes, n, %)	205 (31.5)	358 (28.0)	0.05
Adolescent’s allergic rhinitis history (Yes, n, %)	195 (30.0)	372 (29.1)	0.711
Adolescent’s eczema history (Yes, n, %)	67 (10.3)	168 (13.1)	0.07
Pet ownership (Yes, n, %)	205 (31.5)	358 (28.0)	0.05
Parental atopic history (Yes, n, %)	538 (82.3)	1020 (79.6)	0.087
Parental ever smoking (Yes, n, %)	408 (62.5)	674 (52.6)	< 0.001
Parental atopic history (Yes, n, %)	116 (17.7)	262 (20.4)	0.164
Cooking with fuels (Yes, n, %)	344 (53.0)	648 (50.6)	0.335
Exposure to dampness and/or mould (Yes, n, %)			
*Current exposure*	141 (21.6)	319 (24.9)	0.104
*Exposure during pregnancy*	51 (7.8)	88 (6.9)	0.452
*Exposure during the 1*^*st*^* year of the child’s life*	84 (12.8)	146 (11.4)	0.373

* up to 9 years of education ** university/college/post-graduate studies

**Table 3:** Results from the logistic regression analysis that evaluated the association of indoor moisture environment in the presence of adolescents’ asthmatic history and current asthma symptoms, according to parental educational level (OR, 95% CI*)

	Crude OR	Adjusted OR**	***p*** for interaction		Crude OR	Adjusted OR	***p*** for interaction		Crude OR	Adjusted OR	***p*** for interaction
				Ever had asthma							
Current exposure to dampness and/or mould				Exposure to dampness and/or mould during pregnancy				Exposure to dampness and/or mould during 1^st^ year of life			
*Yes vs. No*	1.73 (1.23–2.41)	1.52 (1.06–2.19)	< 0.001		1.09 (0.75–1.59)	1.15 (0.61–2.17)	0.435		0.73 (0.47–1.14)	1.09 (0.75–1.6)	0.839
				Current asthma							
Current exposure to dampness and/or mould				Exposure to dampness and/or mould during pregnancy				Exposure to dampness and/or mould during 1^st^ year of life			
*Yes vs. No*	1.61 (1.05–2.41)	1.66 (1.1–2.51)	< 0.001		0.84 (0.43–1.65)	0.94 (0.48–1.83)	0.873		0.65 (0.39–1.09)	0.70 (0.42–1.17)	0.727

*OR: Odds Ratio, 95% CI: Confidence Intervals ** adjusted for the confounders: sex, children’s Body Mass Index, parental atopic history, adolescents’ history of allergic rhinitis, adolescents’ history of eczema, pet ownership, parental smoking, having an older sibling, cooking with fuels

**Table 4:** Results from the logistic regression analysis that evaluated the association of indoor moisture environment in the presence of adolescents’ asthmatic history, according to parental educational level

Parental educational level: primary / secondary*									
			Ever had asthma						
	Crude OR	Adjusted OR***	Crude OR		Adjusted OR			Crude OR	Adjusted OR
Current exposure to dampness and/or mould			Exposure to dampness and/or mould during pregnancy				Exposure to dampness and/or mould during 1^st^ year of life		
*Yes vs. No*	2.01 (1.09–3.72)	1.96 (1.05–3.68)		0.96 (033–2.77)		0.77 (0.35–1.70)		0.70 (0.27–1.82)	1.54 (0.93–3.59)
			Current asthma						
Current exposure to dampness and/or mould			Exposure to dampness and/or mould during pregnancy				Exposure to dampness and/or mould during 1^st^ year of life		
*Yes vs. No*	1.71 (0.88–3.32)	1.63 (0.83–3.22)		0.52 (0.21–1.29)		0.54 (0.22–1.36)		0.56 (0.26–1.21)	0.59 (0.28–1.31)
Parental educational level: tertiary**									
Ever had asthma									
	Crude OR	Adjusted ORs		Crude OR		Adjusted ORs		Crude OR	Adjusted OR
Current exposure to dampness and/or mould			Exposure to dampness and/or mould during pregnancy				Exposure to dampness and/or mould during 1^st^ year of life		
*Yes vs. No*	1.60 (1.08–2.39)	1.55 (1.04–2.32)		0.97 (0.33–2.83)		0.71 (0.32–1.58)		0.62 (0.34–1.12)	0.76 (0.52–1.11)
			Current asthma						
Current exposure to dampness and/or mould			Exposure to dampness and/or mould during pregnancy				Exposure to dampness and/or mould during 1^st^ year of life		
*Yes vs. No*	1.66 (1.01–2.71)	1.52 (1.03–2.51)		1.32 (0.47–3.73)		1.49 (0.53–4.33)		0.73 (0.38–1.42)	0.79 (0.41–1.55)

*up to completed 9 years of education ** university/college/postgraduate studies ***adjusted for the confounders: sex, children’s Body Mass Index, parental atopic history, adolescents’ history of allergic rhinitis, adolescents’ history of eczema, pet ownership, parental smoking, having an older sibling, cooking with fuels

## Data Availability

The datasets generated and/or analysed during the current study are not publicly available due to copyright reasons as a part of the Global Asthma Network study but are available from the corresponding author on reasonable request.

## Abbreviations

GAN: Global Asthma Network
SES: Socioeconomic Status
IDM: Indoor dampness and/or mould
BMI: Body Mass Index
SD: Standard Deviation
OR: Odds ratio
CI: Confidence Intervals
Th2: T-helper cell type 2
IgE: Immunoglobulin E
